# The complete chloroplast genome of *Vitis amurensis* Rupr., an economic plant to China

**DOI:** 10.1080/23802359.2019.1678431

**Published:** 2019-10-21

**Authors:** Jun Xie, Xiaojing Guo, Rong Wang, Jing Liu, Yujuan Liu, Gaixia Qiao, Meilong Xu

**Affiliations:** State Key Laboratory of Seeding Bioengineering, Ningxia Forestry Institute, Yinchuan, PR China

**Keywords:** *Vitis amurensis*, chloroplast genome, Illumina sequencing, phylogenetic analysis

## Abstract

The chloroplast (cp) genome sequence of *Vitis amurensis* has been characterized from Illumina pair-end sequencing. The complete cp genome was 161,014 bp in length, containing a large single-copy region (LSC) of 89,239 bp and a small single-copy region (SSC) of 19,067 bp, which were separated by a pair of 26,354 bp inverted repeat regions (IRs). The genome contained 133 genes, including 88 protein coding genes, 37 tRNA genes, and 8 rRNA genes. The overall GC content is 37.4%, while the corresponding values of the LSC, SSC, and IR regions are 35.3%, 31.7%, and 43.0%, respectively. Further, phylogenetic analysis suggested that the *V. amurensis* was sister to *Vitis coignetiae*.

*Vitis amurensis* Rupr., is one of striking wild germplasms of the East Asian *Vitis* spp. It often grows in hillside or ravine on hillsides at elevations of 200–2100 m in the northeast areas, and Hebei, Shanxi, Shandong, Anhui, and Zhegjiang province of China (Chen et al. 1987). This geographical distribution of this species confers their strong tolerance to cold. At present, grapes were mainly made available as a fresh edible fruit and a small part has also been used for making wines in China. However, it has not yet been effectively developed and fully utilized. As one of the important genetic marker sources, chloroplast genomes are widely used for phylogenetic analyses, genetic diversity evaluation, and plant molecular identification.

The total genomic DNA was extracted from dry leaves using a modified CTAB method (Doyle and Doyle 1987) and sequenced based on the Illumina pair-end technology. The voucher specimen was collected in the Yinchuan Botanical Garden (38°28′N, 106°16′E; Ningxia, NW China), and stored at State Key Laboratory of Seeding Bioengineering, Ningxia Forestry Institute (2009PC0928). The filtered reads were assembled using the programme NOVOPlasty (Dierckxsens et al. 2017). The assembled chloroplast genome was annotated using Plann (Huang and Cronk 2015), and the annotation was corrected using Geneious (Kearse et al. 2012). The physical map of the new chloroplast genome was generated using OGDRAW (Lohse et al. 2013). The accurate new annotated complete chloroplast genome was submitted to GenBank with accession number MN398394. The complete chloroplast genome of *V. amurensis* is161,014 base pairs (bp) in length, containing a large single-copy (LSC) region of 89,239 bp, a small single-copy (SSC) region of 19,067 bp, and two inverted repeat (IR) regions of 26,354 bp. The new sequence possesses total 133 genes, including 88 protein-coding genes, 8 rRNA genes, and 37 tRNA genes. Among all of these genes, four rRNA genes (i.e. 4.5S, 5S, 16S, and 23S rRNA), eight protein-coding genes (i.e. *ndhB*, *rpl2*, *rpl23*, *rps7*, *rps12*, *ycf1*, *ycf15,* and *ycf2*), and seven tRNA genes (i.e. *trnA-UGC*, *trnI-CAU*, *trnI-GAU*, *trnLCAA*, *trnN-GUU*, *trnR-ACG*, and *trnV-GAC*) occur in double copies. The overall GC-content of the whole plastome is 37.4%, while the corresponding values of the LSC, SSC, and IR regions are 35.3%, 31.7%, and 43.0%, respectively.

To confirm the phylogenetic position of *V. amurensis*, 37 complete chloroplast genome sequences of Vitaceae were aligned using MAFFT version 7 (Katoh and Standley 2013) and maximum parsimony (MP) analysis was conducted using with PAUP software version 4.0b10 with 1000 bootstrap replicates (Swofford 2003). The MP tree showed that *V. amurensis* was sister to *Vitis coignetiae* (Figure 1).

**Figure 1.**
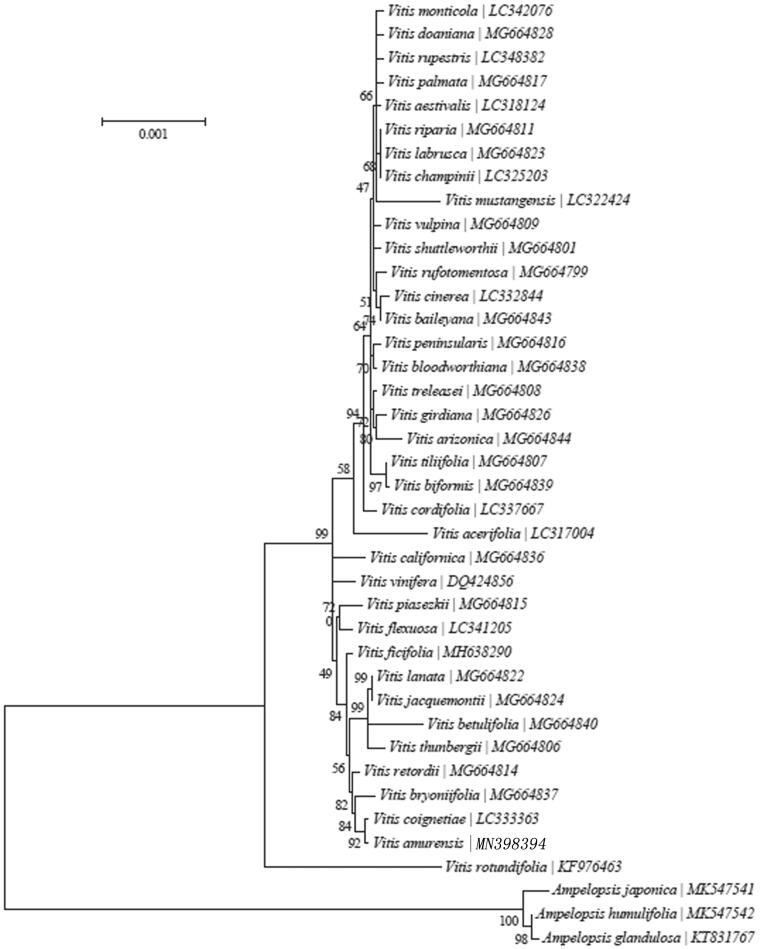
Maximum-likelihood (ML) tree of *V. amurensis* and its related relatives based on the complete chloroplast genome sequences.
